# Osmotic Adaptation by Na^+^-Dependent Transporters and ACE2: Correlation with Hemostatic Crisis in COVID-19

**DOI:** 10.3390/biomedicines8110460

**Published:** 2020-10-30

**Authors:** Danah Muhanna, Shanvanth R. Arnipalli, Shashi B. Kumar, Ouliana Ziouzenkova

**Affiliations:** Department of Human Sciences, The Ohio State University, Columbus, OH 43210, USA; muhanna.2@buckeyemail.osu.edu (D.M.); arnipalli.1@buckeyemail.osu.edu (S.R.A.); kumar.864@osu.edu (S.B.K.)

**Keywords:** angiotensin, tonicity, transporters, virus, thrombosis, hypertension, coagulation, organ failure, inflammation

## Abstract

COVID-19 symptoms, including hypokalemia, hypoalbuminemia, ageusia, neurological dysfunctions, D-dimer production, and multi-organ microthrombosis reach beyond effects attributed to impaired angiotensin-converting enzyme 2 (ACE2) signaling and elevated concentrations of angiotensin II (Ang II). Although both SARS-CoV (Severe Acute Respiratory Syndrome Coronavirus) and SARS-CoV-2 utilize ACE2 for host entry, distinct COVID-19 pathogenesis coincides with the acquisition of a new sequence, which is homologous to the furin cleavage site of the human epithelial Na^+^ channel (ENaC). This review provides a comprehensive summary of the role of ACE2 in the assembly of Na^+^-dependent transporters of glucose, imino and neutral amino acids, as well as the functions of ENaC. Data support an osmotic adaptation mechanism in which osmotic and hemostatic instability induced by Ang II-activated ENaC is counterbalanced by an influx of organic osmolytes and Na^+^ through the ACE2 complex. We propose a paradigm for the two-site attack of SARS-CoV-2 leading to ENaC hyperactivation and inactivation of the ACE2 complex, which collapses cell osmolality and leads to rupture and/or necrotic death of swollen pulmonary, endothelial, and cardiac cells, thrombosis in infected and non-infected tissues, and aberrant sensory and neurological perception in COVID-19 patients. This dual mechanism employed by SARS-CoV-2 calls for combinatorial treatment strategies to address and prevent severe complications of COVID-19.

## 1. Introduction

**Figure 1:**
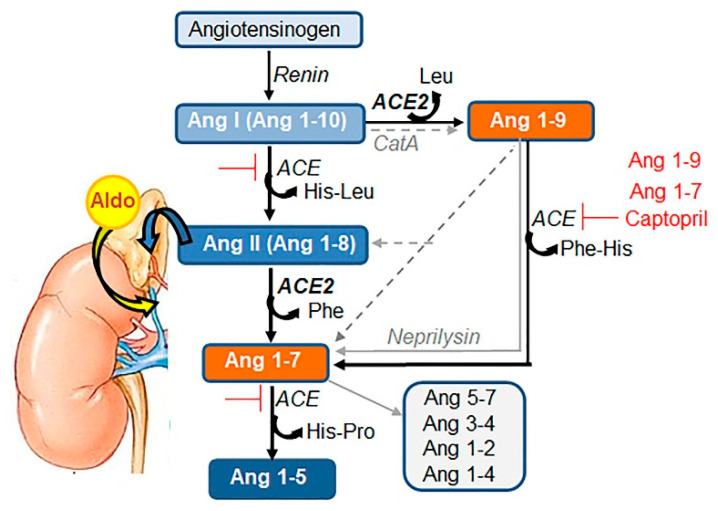
Components of the Renin-Angiotensin System (RAS). Bioactive peptides of angiotensinogen, Ang I or Ang (1–10), Ang II or Ang (1–8), and Ang (1–7) are depicted as rectangular shapes. The cleavage enzymes of renin, angiotensin-converting enzyme (ACE), ACE2, cathepsin A (CatA), and neprilysin are indicated in italics. The central panel shows the canonic RAS pathway. ACE cleaves dipeptide His-Leu, which converts decapeptide Ang I into octapeptide Ang II (blue rectangles). ACE2 cleaves a neutral amino acid, Phe, that converts Ang II into Ang 1–7 (orange rectangles). The alternative pathways by ACE and ACE2 (black arrows) as well as those mediated by other enzymes (gray lines) are shown on the right. Captopril is synthetic and Ang 1–7 and Ang 1–9 are natural inhibitors of ACE (red lines). Ang 1–7 inhibition presents a classical feedback mechanism for physiological control of Ang II levels in circulation. One example of the convergence of Ang II with other endocrine pathways for the regulation of blood pressure is shown on the left. Circulating Ang II initiates the production of aldosterone (Aldo) from cholesterol in the adrenal cortex.

**Figure 2:**
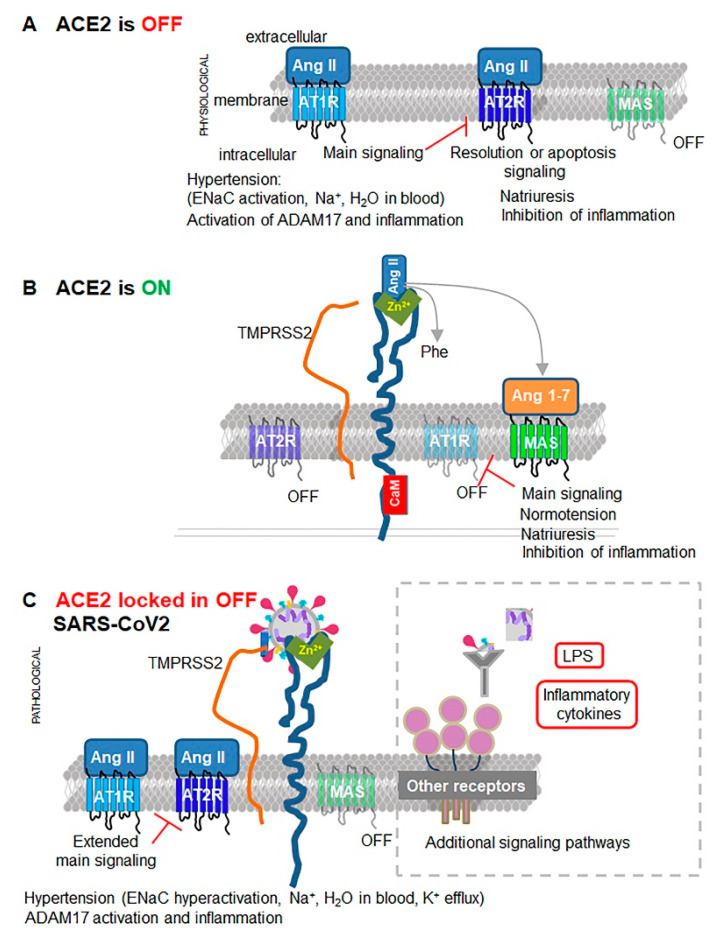
Schematic regulation of ACE2-dependent pathways under physiological conditions and during SARS-CoV-2 infection. (**A**) Main signaling in the absence of ACE2 when it is not expressed, or its activity is inhibited (ACE2 is ‘OFF’). Circulating Ang II binds to its high affinity G-protein-coupled receptor AT1R. Main AngII/AT1R signaling mediates vasoconstriction, NADPH oxidase activation and reactive oxygen species (ROS) formation, hypoxia induction (HIF1α), activation of MMPs, including ADAM17 and pro-inflammatory cytokines, Na^+^/H_2_O retention from extracellular fluids into blood, hypertension, hypertrophy, activation of sympathetic nerves and other effects. Ang II/AT1R receptor can suppress angiotensin 2 receptor (AT2R, red lines) expression in the initial stages of inflammation, but this suppression is alleviated by interferon regulatory factor 1 (IRF-1). Ang II also activates AT2R, sometimes in complex with different proteins, which opposes the effects of Ang II/AT1R and resolves of stress, inflammation, and apoptosis. Under specific conditions, Ang II/AT2R response could mimic Ang II/AT1R possibly by involving further cleavage of Ang II. MAS receptor remains inactivated (OFF) without ligand Ang (1–7), produced from Ang II. (**B**) The main signaling in the presence of catalytically active ACE2 (ACE2 is ‘ON’). Activated ACE2 is shown as an integral protein with an active catalytic site containing Zn^2+^ in the ectodomain (green V shape). ACE2 is stabilized in a catalytically active conformation by calmodulin (CaM, red rectangle) bound to an intracellular domain of ACE2 that prevents cleavage of ACE2 by proteases ADAM17 (not shown) or TMPRSS2 (orange line shape). ACE2 cleaves Ang II, leaving both AT1R and AT2R inactive (OFF). ACE2 cleavage produces Ang 1–7, which binds to MAS receptor and elicits signaling that opposes Ang II/AT1R effects and inhibits AT1R expression (red lines). (**C**) SARS-CoV-2 (circular shape) binds to the catalytic site of ACE2 for entry. SARS-CoV-2 has also acquired a new sequence that mimics the furin cleavage site of human ENaC (blue cylinder inside viral shape). TMPRSS2 is an inhibitor of ENaC; however, in the presence of SARS-CoV-2, it possibly binds to ENaC mimetic site for cleavage of viral spike proteins for replication. Host ENaC remains active (not shown). The resultant pathological response becomes Ang II-centered and cannot be resolved in the absence of Ang (1–7)/MAS signaling due to the functional hindrance of ACE2 by SARS-CoV-2 over a supraphysiological period of time (locked in ‘OFF’). These pathways initiate the pathologies seen in COVID-19 patients (discussed below). Additional inflammatory events triggered by viral mRNA, viral protein and its complexes with host antibodies, host’s inflammatory responses, and probable bacterial co-infections and their lipopolysaccharide (LPS) will be mediated by other receptors (gray dashed square). Although important, these responses will be secondary to viral binding to ACE2 and will not be discussed in this review.

Viruses have evolved to hijack specific multifunctional proteins that assist in viral entrance and subsequent viral proliferation, while simultaneously disabling the host’s metabolic responses and defense mechanisms. This strategy is employed through Severe Acute Respiratory Syndrome Coronavirus (SARS-CoV) and SARS-CoV-2 beta coronaviruses’ binding and utilization of angiotensin-converting enzyme 2 (ACE2) (reviewed in [1]). ACE2 is a type I integral membrane protein expressed in epithelial and vascular endothelial cells in the lungs, intestines, heart, kidneys, brain, and other organs (reviewed in [2]). The classical function of ACE2 is the cleavage of peptide hormone angiotensin II (Ang II) within the renin-angiotensin system (RAS) (Figure 1, reviewed in [3,4]). Ang II binds to Ang II receptor type I (AT1R) to control blood pressure, and multiple other responses [1] in barrier tissues expressing ACE2 (Figure 2A) [2]. The resulting ACE2 cleavage product Ang (1–7) mediates vasodilatory and anti-inflammatory effects through the MAS receptor (alias Mas1, MasR; reviewed in [5,6]) (Figure 2B), which counteracts the damaging effects of Ang II/AT1R (reviewed in [7]). Surprising findings in mice deficient in ACE2 (Ace2^−/−^) reveal that ACE2 does not directly control blood pressure [8] and has a gamut of functions that cannot be attributed merely to the cleavage of Ang II [9,10]. Emerging studies suggest that several new functions attributed to ACE2 depend on interactions with other proteins [11,12,13,14], which are the focus of this review.

The highly pathogenic SARS-CoV-2 coronavirus, with a lower affinity but higher efficacy of interaction with ACE2 than SARS-CoV [12,15,16], is now a global public health threat exceeding 45 million cases worldwide (updated online [17]). ACE2 catalytic site is an entry route for SARS-CoV-2, which impacts the cleavage of Ang II (Figure 2C) resulting in a shift in an inflammatory response from defensive and beneficial, to detrimental to the host (reviewed in [18]). The critical pathophysiological scenarios of severe morbid COVID-19 manifestations (reviewed in [19]), particularly venous microthrombosis, did not occur under severe inflammatory conditions mediated by other viruses [20] and reaches beyond inhibition of the catalytic function of ACE2. In this review, we summarize recent findings regarding an ACE2-centered supramolecular complex and propose the critical role of ACE2 in the regulation of cell volume in the context of coagulation, which becomes apparent after the disrupting impact of SARS-CoV-2 infection. We outline distinct pathways triggered by a unique SARS-CoV-2 structural site [21] responsible for effective viral transmission and severe pathogenicity of COVID-19. The goal of this theoretical paper is to provide a framework that assists researchers across multiple fields in experimentally examining both the functions of ACE2 in network with other proteins, and the malfunction of this network in the presence of SARS-CoV-2.

## 2. ACE2 Structure and Catalytic Site

ACE2 (805 amino acids (aa), 92.5 kD [22,23]) has three principal domains: (1) N-terminal extracellular domain or ectodomain, (2) membrane-spanning domain, and (3) C-terminal intracellular domain. Ectodomain regions that have structural and functional similarity to other molecules are commonly termed ‘domains’, e.g., collectrin-like domain. Each of these functional ‘domains’ or sites have a specific or opportunistic partner that alters its function in conjunction with their physiological and pathological functions, as well as in the context of SARS-CoV-2.

The most characterized extracellular site of ACE2 is a single, conserved zinc metalloprotease consensus HEXXH that efficiently cleaves a neutral aa from the C-terminal of Ang II (alias Ang 1–8) to produce Ang 1–7 and phenylalanine [22]. ACE2 also cleaves other substrates [24] (Box 1). ACE2 cleavage of Ang I provides an alternative cleavage mechanism in animals treated with ACE inhibitors [23] and is relevant for hypertensive patients treated with these compounds. Apelin–13 and dynorphin A are additional high-affinity substrates for ACE2, which exhibit 91% and 60% less activity compared to Ang II (100%), respectively [24]. Catalytically active ACE2 (~120 kD) is glycosylated [22]; however, the requirement of glycosylation for this activity has yet to be examined. Enzymatic activity of ACE2 is not restricted to cells expressing ACE2, since the catalytic ectodomain of ACE2 can be released into circulation following cleavage by proteases ADAM17 and TMPRSS2 [25]. Enzymatic function of ACE2 assures the balance between Ang II and Ang 1–7 [22] within RAS (Figure 1) and apelin pathways [24].

Box 1 **Characteristics of ACE2 catalysis.**

- Catalytic site: HEXXH (Alias: HEMGH, or benzylsuccinate insensitive carboxypeptidase, peptidase domain [22]).
- Low affinity substrates [23,24]: angiotensin I (Ang 1–10), des-Arg bradykinin, β-casomorphin, ghrelin, neurotensin1–13, neurotensin1–8 kinetensin.
- Optimal catalytic conditions [24]: Zn^2+^, pH 6–8; chloride anion (1 M NaCl) binds to Arg514.
- Inhibitors: EDTA [23].

## 3. Effects of Ang II and Its Downstream Target ENaC on Osmosis, Hypertension, and Swelling

**Figure 3:**
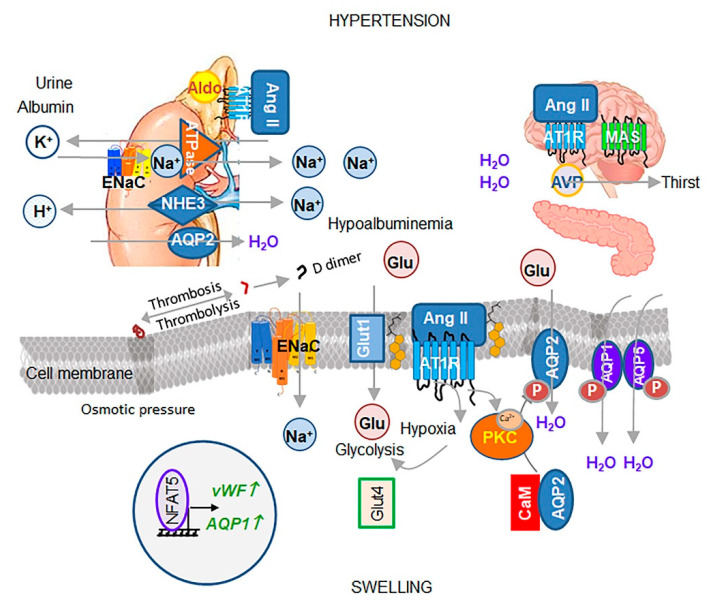
The principal components of osmotic regulation by Ang II (ACE2 OFF). In the cholesterol-rich membrane (yellow structures) of barrier cells (specified below), Ang II/AT1R activates classical and novel protein kinase C (PKC) and mobilizes Ca^2+^ from internal stores. Ca^2+^ binds to calmodulin (CaM), resulting in disassembly of AQP2 and CaM complex retaining AQP2 in the cytosol of cells expressing AQP2 (cortical and medullary renal collecting ducts, pancreatic islets, fallopian tubes, peripheral nerves [33], as well as lymphatic endothelium [34]). PKC phosphorylation of AQP2 (P-AQP2) and Ca^2+^-activated cytoskeleton translocate to membrane P-AQP2 for stimulated water influx. Epithelial or endothelial cells express AQP1 and AQP5 (reviewed in [35]). PKC [36] can activate AQP1, whereas a transient receptor potential vanilloid 4 (TRPV4)-triggered [37] mechanism has been proposed for AQP5-mediated water flux reducing lung edema, potentially via PKC activation [38]. Ang II/AT1R mobilizes ENaC for Na^+^ influx and its transit through the cell with the help of Na^+^/K^+^ ATPase (detailed in Figure 4). Ang II/AT1R inhibits insulin secretion and maintains GLUT4 in the cytosol, which creates a deficit of organic osmolytes in the cell. Glucose influx is mediated via the GLUT1 transporter for glycolytic energy production under Ang II/AT1R-stimulated hypoxia via HIF1α transcription factor. The deficit of organic osmolytes and Na^+^, and an excess of water inflow, leads to cell swelling. Changed cellular tonicity activates NFAT5 transcription factor and its target genes vWF and AQP1; vWF, which stimulate perpetuate microthrombi formation followed by the breaking down of fibrin into D-dimer. In the adrenal gland, Ang II/AT1R induces aldosterone secretion, which activates ENaC-dependent reabsorption of Na^+^ into blood in exchange for K^+^ in the kidney. NHE3 reabsorbs Na^+^ from urine in exchange for H^+^. These transporters establish hypernatremia and hypokalemia in the blood. Hypoalbuminemia in blood compensates for cellular hypotonicity. Ang II/AT1R also stimulates arginin vasopressin (AVP), increasing stimulated thirst, while MAS remains inactivated. Ang II-centric events lead to hypertension treated with ACE inhibitors and AT1R blockers.

Ang II/AT1R is one of the major mechanisms controlling water and Na^+^ concentrations in blood, which contributes to increase in extracellular fluid volume, leading to hypertension (Figure 3) (reviewed and proposed in [26]). Under additional stress, in coordination with hormones (Box 2) Ang II regulates reabsorption of water and Na^+^ in the kidney epithelium. In contrast, lung and vascular tissues have an autonomous regulation of Na^+^ reabsorption from extracellular spaces into epithelium by cellular proteases, including TMPRSS2 and furin (reviewed in [27]).

Box 2 **Ang II/AT1R-regulated hormones.**

- Aldosterone in the kidney cortex [28]; stressor: high salt.
- Vasopressin (or arginine vasopressin) in the hypothalamus [29]; stressor: water deprivation.

Direct targets of Ang II/AT1R on Na^+^ reabsorption [30,31] were identified in the Ang II infusion model of hypertension [28]. Although multiple other transporters and claudins contribute to this process, systematic studies in male and female rodents [28,31] revealed a central contribution of an amiloride-sensitive epithelial Na^+^ transporter (ENaC) in Na^+^ reabsorption from urine and other extracellular fluids [28,31] (Figure 3). Genetic deficiency in αENaC, βENaC, or γENaC in the lung results in a morbid electrolyte imbalance leading to lung inflammation and death (excellently reviewed in [32]).

**Figure 4:**
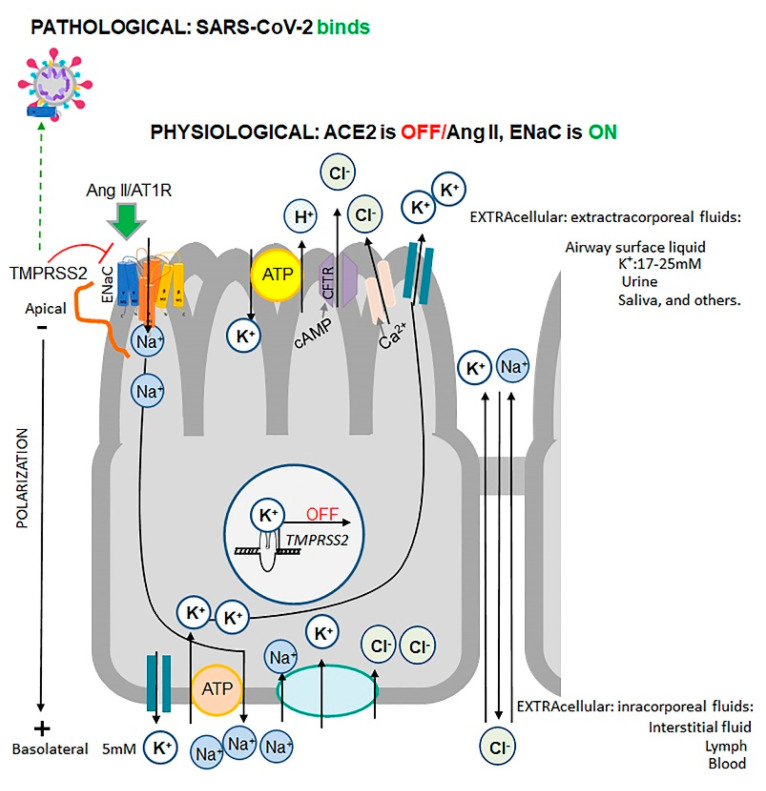
Pathophysiological regulation of ENaC in barrier cells, such as type II pneumocytes expressing *ACE2*, *TMPRSS2* [48], and *ENaC* [49]. These cells are a validated target of SARS-CoV-2 and exhibit viral particles in all investigated post-mortem lung tissues of COVID-19 patients [50], though other barrier cells, including ileal absorptive enterocytes, ciliated cells, and nasal goblet secretory cells, can also be infected [48]. Endothelial cells express ACE2, but not TMPRSS2 in the post-mortem lung tissues from COVID-19 patients [51]. Endothelial cells do not contain SARS-CoV-2 particles [50] but exhibit osmotic malfunctions: swelling, rupture, microthrombosis, and/or necrosis in these tissues [20,50], that may involve ENaC expressed in these cells [41]. Physiological regulation of ENaC activity is facilitated via feedback mechanism. In the ‘OFF’ state of ACE2, Ang II/AT1R (green arrow) induces mobilization and expression of the αβγENaC (blue, yellow, and orange shapes) to the apical side of barrier cells. Na^+^ follows a gradient to the basolateral side where Na^+^/K^+^ ATPase transporters mediate efflux of Na^+^ into the blood in exchange for K^+^. Na^+^/K^+^ ATPase transporters, cystic fibrosis transmembrane conductance regulator (CFTR), and Na^+^-K^+^-Cl^−^ cotransporter 1 (NKCC1) are expressed in pneumocytes and regulate alveolar fluid balance [52]. This Na^+^ flow establishes a positive transcellular current (polarization) of ~2.7 ± 0.5 μA/cm^2^. The K^+^ follows a gradient to the apical side where its efflux is mediated by multiple transporters leading to a 3–5-fold higher concentration of K^+^ in airway surface fluid (15–27 mM K^+^), compared to blood (5 mM K^+^). TMPRSS2 (orange line shape) binding inhibits ENaC; however, TMPRSS2 is suppressed by K^+^ ions bound to G quadruplex in the promoter region (nucleus). Intracellular loss of K^+^ activates TMPRSS2 expression creating a potential feedback loop for inhibition of ENaC activity. Pathological regulation. The furin cleavage site on SARS-CoV-2 is homologous to human ENaC and therefore, can provide an alternative site for binding of TMPRSS2, which can cleave SARS-CoV-2 for replication. Host’s ENaC remains activated without inhibition by TMPRSS2.

In tissues expressing ENaC (described in Box 3), ENaC functions for ~20 min [27] and requires Ang II for its expression and mobilization to the apical membranes [30,31] (Figure 4). Following uptake by ENaC, Na^+^ follows an electrochemical gradient to the basolateral membrane, where Na^+^/K^+^ ATPase transporters mediate the efflux of Na^+^ to the blood in exchange for K^+^ [39] (Figure 4). This action establishes a positive transcellular current ~2.7 ± 0.5 μA/cm^2^ from apical-to-basolateral sides, known as cell polarization [39]. The electrochemical gradient favors apical cellular K^+^ efflux through K^+^ channels and the apical H^+^/K^+^ pump [39]. These effects establish urinary K^+^ excretion in the kidney, leading to a decrease of K^+^ and an increase of Na^+^ concentrations in the blood [27]. In the lungs, ENaC in conjunction with other transporters establishes a 5-fold greater K^+^ concentration in the extracellular airway surface liquid compared to basolateral K^+^ concentrations [39] (Figure 4).

Box 3 **ENaC composition and expression.**

Expression of subunits: α, β, γ, and δ: αβγENaC in kidney, lungs, GI tract, testes, salivary duct [40], vascular endothelial cells [41], other barrier cells δENaC subunit in neurons, heart, and pancreas.

Physiological ENaC activity is regulated by a feedback mechanism dependent on K^+^ concentrations (Figure 4). A K^+^-stabilized secondary guanine quadruplex element (G-quadruplex) of DNA [42] is found in promoters of genes involved in the inflammatory response, including proliferative c-Myc [43], anti-apoptotic BCL1, angiogenic VEGF, TMPRSS2 [44] and others (reviewed in [45]). Decreases in intracellular K^+^ concentrations alleviate the silencing of the TMPRSS2 promoters and increase gene expression [44] (Figure 4). TMPRSS2 binding to ENaC inhibits ENaC [46], which prevents loss of K^+^. TMPRSS2 is a type II transmembrane serine protease [47] that also cleaves ACE2 [25]; however, it is unknown if either the misfolded or oxidized form of ACE2 is the preferred substrate for TMPRSS2. Physiological Ang II concentrations and TMPRSS2 feedback adjust ENaC levels and its activity with functions of other transporters for an ionic osmotic balance between extracellular and intracellular fluids in the kidneys, lungs, and GI tract.

The progressive urinary loss of K^+^ cannot be compensated by recruitment of other transporters, NCC [53], Kir4.1, Na^+^/H^+^ exchanger (NHE3) [28], or AT2R in nephrons [54], which leads to severe hypertension [28,30]. Overactivation of ENaC is lethal. In genetic studies, overexpression of prostasin, activating ENaC, leads to electrolyte imbalance and hypertension in mice [55]. Overactivation of ENaC in the lung epithelium in mice deficient in ubiquitin ligase Nedd4L (Nedd4L^−/−^) also results in lung inflammation, fibrosis, and death, which could be rescued by administration of nasal amiloride inhibitor of ENaC [56]. Although Nedd4L has different targets (reviewed in [57]), the experiment with amiloride highlights the chief role of ENaC in lung inflammation. In humans, a genetic overactivation mutation in βγENaC [58] causes Liddle syndrome. In this disease, excessive reabsorption of Na^+^ by overactive βγENaC leads to expanded extracellular fluid volume that suppresses renin and aldosterone causing severe hypertension in childhood accompanied by hypokalemia and high pH (metabolic alkalosis) [27]. TMPRSS2 inhibition of ENaC [46] provides a fine-tuning of electrolytes and osmolytes, and protects from hypokalemia.

Overproduction of Ang II, aldosterone, vasopressin, and/or inflammatory cytokines (reviewed in [59]) compromises intracellular osmotic stability [60] by diminishing the supply of organic osmolytes, particularly glucose. Ang II reduces secretion of insulin, and glucose uptake by GLUT4 and SGLT1 [61] (Figure 3). Instead, Ang II upregulates GLUT1, transporting glucose to meet energy demands. The loss of intracellular Na^+^ and organic osmolytes in the cell does not necessarily alter the concentration of these metabolites in circulation. Albumin compensates for reduced intracellular concentrations of ions and organic osmolytes in order to achieve Donnan equilibrium [62]. The reduction of 1 g/dL albumin in serum (reviewed in [63,64]) corresponds to a reduction of 2 mmol Na^+^/L [62]. Ang II-mediated stress [65] is accompanied by hypoalbuminemia, which is a diagnostic marker for intracellular hyponatremia [66].

Ang II stress is exacerbated by changes in water homeostasis. Ang II/AT1R engages Gαq/11, which in turn activates phospholipase C (PLC) [67] leading to Ca^2+^ release (reviewed in [68]) and activation of conventional and novel PKC types [68] (Figure 3). PKC-mediated phosphorylation of aquaporins AQP1 [69] and AQP5 [70] leads to their translocation to the membrane surface for stimulated water influx overriding tonicity and concentration of organic osmolytes [69]. In the kidney, Ang II/AT1R induces a similar PKC signaling cascade, including calmodulin (CaM) and PKC activation, which increases AQP2-dependent water reabsorption from urine into the blood [71]. Ang II-dependent release of arginine vasopressin stimulates thirst and additional water reabsorption in the kidney by AQP2 [71] (Figure 3). Arginine vasopressin overrides the physiologic regulation of tonicity in cardiac muscles under chronic heart failure conditions by stimulation of water influx through AQP2 in the renal collecting duct [72], which induces hyponatremia [73]. Ang II/AT1R in the central nervous system (CNS) appears to govern cardiac effects, hence overexpression of human ACE2 in mouse neurons was sufficient to prevent cardiac tissue remodeling and associated blood pressure for at least 14 days [74]. Cumulatively, the deranged osmotic balance and water influx often contribute to cellular hypertrophy and swelling in the presence of Ang II [74].

The regulation of extracellular osmotic responses was validated in the loss-of-function AT1R^−/−^ [29] and Ace^−/−^ mice with reduced Ang II production. In these mice, deficient Ang II/AT1R signaling reduced blood pressure by decreasing urine osmolality (−64%) and increasing urinary water content [29]. Given these established functions of Ang II, the gain of Ang II function in Ace2^−/−^ mouse model was created [8] and was expected to highlight blood pressure regulation by Ang II. However, the phenotype of Ace2^−/−^ mice revealed that ACE2 function in osmotic balance reaches beyond the traditional cleavage of Ang II.

## 4. ACE2 Partnership with Neutral aa Transporter B^0^AT1

The identification of an integral role of ACE2 in the assembly of a multiprotein complex [75] marks the beginning of the quest for additional functions of this protein. ACE2 is an obligatory partner of Na^+^-dependent B degrees-like neutral aa transporter (B^0^AT1, gene Slc6a19) in the intestine, where Ace2 deficiency abolishes expression of Slc6a19 [10]. B^0^AT1 transports neutral aa (Box 4) and Na^+^, leading to membrane depolarization [11].

Box 4 **Neutral aa (monoamino monocarboxylic aa).**

Alanine, asparagine, citrulline, glutamine, histidine, isoleucine, leucine, phenylalanine, serine, threonine, tyrosine, tryptophan, and valine.

**Figure 5:**
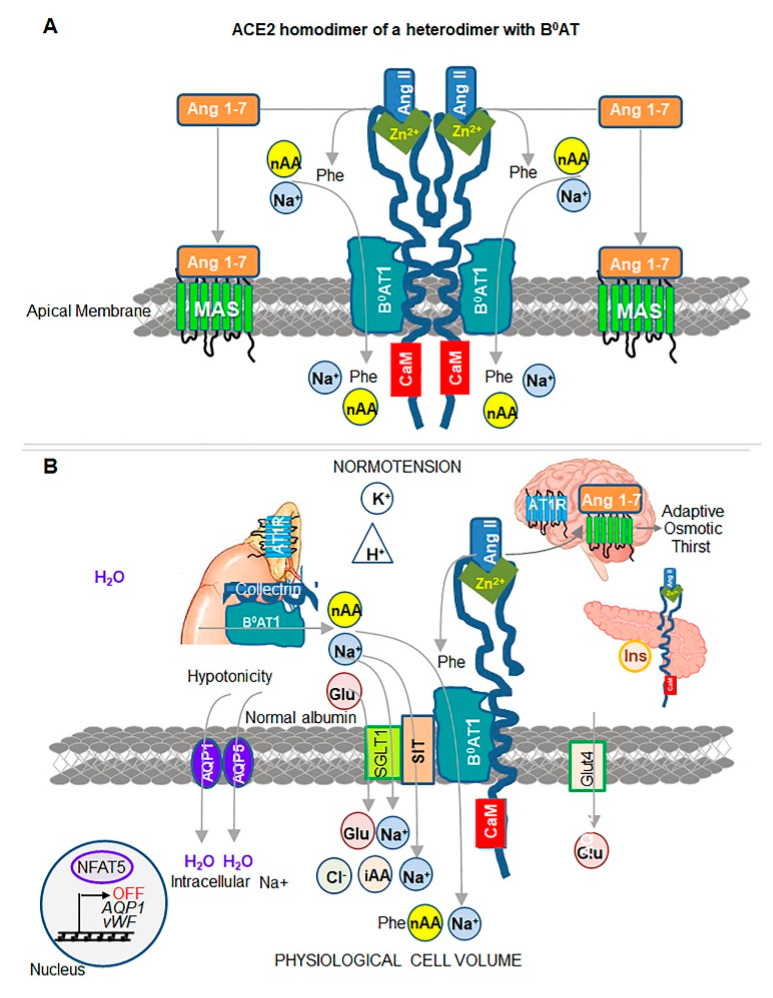
Cumulative function of ACE2 complex with Na^+^-dependent transporters. (**A**) Schematic presentation of enzymatically active ACE2 homodimer undergoing heterodimerization with sodium-dependent transporter B^0^AT1 (SLCA19) to stabilize this complex, based on cryo-electron microscopy structure [12]. ACE2 homodimerization is mediated by polar interactions and disulfide bridge formation. ACE2 is stabilized by CaM to avoid shedding. B^0^AT1 interacts with extended collectrin-like domain of ACE2. B^0^AT1 transports neutral amino acids (nAA including Phe cleaved from Ang II) across the apical membranes of small intestine, lungs and other organs, excluding the kidneys. One Na^+^ is co-transported per amino acid. (**B**) ACE2 complex with Na^+^-dependent transporters contribute to regulation of physiological cell volume and normotension in blood. For clarity, here and below, the ACE2 dimer has been shown as a monomer. ACE2/B^0^AT1 complex also includes other Na^+^-dependent transporters, such as SIT for imino acids (iAA) and Cl^−^ and SGLT1 for glucose (Glu). In addition to their metabolic value, these nAA, iAA, and glucose serve as organic osmolytes, which maintain osmolality together with Na^+^ ions. Water influx is mediated by aquaporins (AQP1 and AQP5) in response to hypotonicity. Insulin secretion is controlled by pancreatic activity of ACE2/B^0^AT1 complex that increase glucose intake by glucose transporter 4 (GLUT4). The balanced levels of organic osmolytes, ions, and water sustain physiologic cell volume, thereby NFAT5 and its target genes vWF and AQP1 remain inactive (OFF). In the kidney, B^0^AT1 functions in a complex with collectrin, where it reabsorbs nAA and Na^+^ from urine. Ang (1–7)/MAS signaling in the brain regulates adaptive osmotic thirst [76]. Unliganded AT1R receptor is inactive. The concentration of water, albumin, K^+^, and H^+^ is in the physiologic range. Physiological cell volume is accompanied by normotension on blood when ACE2 complex is intact.

Its partnership with either collectrin (Alias TMEM27, gene Slc6a18) or ACE2 in most tissues is required to elicit a 10-fold higher activity of B^0^AT1 [10] due to enhanced mobilization and expression of this protein. A complex is formed between ACE2′s collectrin-like domain and B^0^AT1 [12]; then, two of these heterodimers form a dimer [12] (Figure 5A). Disruption of this complex in Ace2^−/−^ mice severely impairs absorption of dietary neutral aa, muscle composition weight, growth, and development on a regular diet, and these abnormalities are exacerbated on low-protein diets [9].

Collectrin is an obligatory partner of B^0^AT1 only in the kidney [77], where deficiency in collectrin manifests with severe neutral hyperaminoaciduria [77]. Loss-of-function mutations of B^0^AT1 [78] can cause one or multiple symptoms of the autosomal recessive Hartnup disorder (OMIM 234500) [78,79], particularly aminoaciduria, stunted growth, and development [75]. In this disease, the deficient transport of tryptophan for the synthesis of niacin can manifest as dermatitis and/or cerebellar ataxia [78,79] resembling pellagra. Notably, these symptoms also develop in patients with mutations (e.g., R240Q) [10,11,78], which spare the transport function of B^0^AT1, but, however, disable the formation of the ACE2/B^0^AT1 complex [11,12]. Thus, the interaction between B^0^AT1 and ACE2 is critical for functional competence of both molecules. 

The uptake of dietary and cleaved neutral aa together with Na^+^ by B^0^AT1 (Figure 5A) provides both nutrients and organic osmolytes that regulate physiological cell volume [60]. Data support clustering of ACE2/B^0^AT1 with two other osmolyte transporters [75,80] (Figure 5B). (1) Na^+^ and Cl^−^-dependent imino acid transporter SIT1 (gene Slc6A20) interacts with ACE2 and B^0^AT1 [75] in human and murine intestine [80]. Genetic deficiency of SIT1 leads to iminoaciduria in mice [75] and gains importance in humans in the context of severe COVID-19 complications (discussed in Section 6). (2) Na^+^-dependent glucose transporter SGLT1 (gene Slc5a1) interacts with B^0^AT1 to control 40% of Na^+^-dependent uptake of glucose [75] (Figure 5B). Studies in Ace2^−/−^ [81] and Slc6A19^−/−^ mice [75,82] revealed the role of ACE2/B^0^AT1 complex in expression and secretion of insulin [75,83,84], which is diminished in Ace2^−/−^ mice [81,84] and Slc6A19^−/−^ mice [75]. ACE2, B^0^AT1, SIT1, and SGLT1 cluster assembles into the supramolecular metabolic and signaling hub, which elicits a proportional influx of Na^+^ and organic osmolytes. The increase in tonicity promotes a physiologic influx of water via aquaporins, including AQP1 [69] and AQP5 [70], establishing cell volume. The dependence of B^0^AT1, SIT1, and SGLT1 on Na^+^ (Figure 5B) indicates that the additional and perhaps, main advantage of supramolecular complex formation, is in the regulation of physiological cell volume that counteracts the swelling responses of Ang II (Figure 3).

## 5. Evidence of ACE2-Dependent Regulation of Osmolality, Cell Volume, and Susceptibility to Thrombosis

The phenotype of Ace2^−/−^ mice provides evidence of the role of ACE2′s regulating intracellular osmolality. Ace2^−/−^ mice lack Ang II cleavage [8] and are also deprived of other anti-hypertensive peptides, such as Ang (1–7) [85,86] and Ang (1–9) [87] (Figure 3). Nonetheless, despite high Ang II levels [8], Ace2^−/−^ mice have normal blood pressure [8]. This finding appears paradoxical, if other phenotypic manifestations in Ace2^−/−^ mice described in the fundamental work by Verrey and coworkers [9], are not considered. Osmotic adaptation was severely compromised in these mice and was dependent on protein content in the diet. Ace2^−/−^ mice retained significantly higher content of water in tissues (147%) vs. WT (100%) on a diet with normal protein content [9], in agreement with Ang II-stimulated water retention by Ang II. On a low-protein diet, the water content was decreased in WT (89%); however, it continued to increase in Ace2^−/−^ mice (213%), suggesting that the levels of organic osmolytes were dependent on ACE2 and likely on its interaction with B^0^AT1 and SIT1. The deregulation in organic osmolyte levels was pronounced in urine. Osmolality was decreased to 56% in urine of Ace2^−/−^ mice vs. WT (100%) on a normal protein diet. A low-protein diet reduced osmolality to 40.5% in WT mice and to 16% in Ace2^−/−^ mice compared to the osmolality in WT mice fed a diet with normal protein content [9]. These changes in urinary osmolality may depend on reduced intestinal absorption of neutral aa in Ace2^−/−^ mice with a dysfunctional ACE2/B^0^AT1/SIT1/SGLT1 cluster [9]. The osmotic deregulation in tissues of Ace2^−/−^ mice is blunted, due to the partial compensation by the collectrin/B^0^AT1 complex [10], particularly in the kidney [75] (Figure 3). A full view on the regulation of intracellular osmolyte concentrations could be obtained in tissue-specific knockout models lacking both Ace2 and collectrin. Cardiac tissues naturally express low levels of collectrin [88], and manifest with early deregulation of myocyte size and cardiac hypertrophy in Ace2^−/−^ mice [89]. The distinct osmotic mechanism in cardiomyocytes is supported by incomplete inhibition of this process by AT1R blocker olmesartan [89], or Ang (1–7)/MAS [90], thus, arguing against mechanisms related to enzymatic cleavage of Ang II. The Ang II-independent effect on hypertrophy was also corroborated by findings in mice with cardiac-specific expression of ACE [91]. Ang II production in the hearts of these mice did not increase cardiac hypertrophy over the effects seen in WT after aortic binding [91]. The expended volume of Ace2^−/−^ cardiomyocytes was not compensated by other conventional mechanisms, including an act of active volume regulation [92], as well as transport system A [60] or amino acid antiporter SLC1A5 (ASCT2) [93]. In agreement with the osmotic mechanism, dysfunctional transport of aa by B^0^AT1 [82], stunting mTORC1 activation [94], provokes cardiac hypertrophy. The interaction of ACE2 with B^0^AT1 might also be at work for regulation of osmolality in humans, given a rare SLC6A19-MS7 minisatellite polymorphism associated with essential hypertension [95]. Collectively, these data appear to support a requirement of both ACE2, B^0^AT, and probably, SIT1/SGLT1 for signaling and adaptive regulation of osmolality and cell size (Figure 5B).

Depletion of intracellular aa activates tonicity-regulated transcription factor NFAT5 (or nuclear factor of activated T cells Alias: TonEBP) [96], triggering production of von Willebrand factor (vWF) [97], AQP1 [98] and cyclooxygenase 2 (COX2) [98] in vascular endothelial cells [97] (Figure 3). Disbalance between extracellular and intracellular glucose concentrations can also upregulate NFAT5 [98]. This osmotic instability-mediated release of vWF perpetuates microthrombosis, which is followed by thrombolysis increasing D-dimer concentrations in the blood [97]. The osmotic instability precedes vascular thrombosis in capillaries triggered by an additional stressor. Unlike local inflammation-induced arterial thrombosis, osmotic thrombosis affects multiple endothelial cells with deranged intracellular tonicity and can occur simultaneously in multiple capillaries of several organs. ACE inhibitors and AT1R blockers are not efficient in prevention of venous thrombosis dependent on Ang II. The correction of impaired intracellular tonicity provoking high circulating D-dimer levels with organic osmolytes (Figure 5B) may provide a protective strategy against progression of this unstable state to microthrombosis. 

## 6. Disruptive Viral Partners ACE2 Catalysis

Coronaviruses SARS-CoV, HCoV-NL63, and SARS-CoV-2 have evolved as disruptive opportunistic binding partners of ACE2 [99,100,101]. SARS-CoV and SARS-CoV-2 share 80% amino acid sequence identity [101] and use a principally similar mechanism for cell entry that requires both ACE2 [101] and TMPRSS2 [102] (Figure 2C, Box 5). The S1 subunit in the viral envelop spike S1/S2 protein interacts with the catalytic site of ACE2 [101] via a viral receptor binding domain [102]. TMPRSS2-mediated cleavage at the S1/S2 and the S2′ sites is indispensable for infectivity in most cells; except for human lung cancer Calu-3 cells [102] that utilize endosomal cysteine proteases cathepsins B/L (Cat B/L) [103] or proprotease furin [104]. Both viruses attack by binding to the catalytic domain of ACE2 with similar affinity [12,105]. SARS-CoV-2 has a larger interface for interaction with the human ACE2 [12,105] than SARS-CoV; however, SARS-CoV-2 acquired a new furin cleavage site sequence that underlies its distinct pathogenicity and infectivity in the context of interaction with TMPRSS2 (discussed next).

Box 5 **Components of SARS-CoV-2 invasion mechanisms.**

**New structural sequence on SARS-CoV-2:** Furin cleavage site [104,106] homologous to human ENaC furin cleavage site [21].

**SARS-CoV-2 binding for entry:** SARS-CoV-2 binds to catalytic side of ACE2 or ACE2 complex with B^0^AT1 [12].

**SARS-CoV-2 cleavage:**

- Extracellular: Host’s principal protease TMPRSS2 cleaves S1-S2 proteins of SARS-CoV-2 [102,104,106].
  Relevant physiological functions of TMPRSS2: (1) ACE2 cleavage and inactivation [25],
                       (2) ENaC inhibitor [46].
- Intracellular proteases: Furin [104,106,107].
  Relevant physiological functions of furin: (1) ENaC cleavage and activation [108].
- Alternative proteases: Cathepsins B/L (Cat B/L) [103].

**Principal SARS-CoV-2-infected cells types:** pneumocytes type II [50] (Express ACE2, TMPRSS2, furin, ENaC [48,49].

**Additional SARS-CoV-2-infected cells types:** macrophages [50], as well as enterocytes, ciliated cells, and nasal goblet secretory cells [48].

The most revealing aspect of a recent cryo-electron microscopy in vitro study [12] is that a stable SARS-CoV-2 interaction is achieved by binding to either ACE2 homodimer or an ACE2 dimer of heterodimers in complex with B^0^AT1 (Figure 5A). The dimerization of the cytosolic domain of ACE2 exists in open and closed ‘claw-like’ conformations [12]. SARS-CoV-2 can bind only to the closed conformation. This study implies that SARS-CoV-2 may only have a narrow window of opportunity to bind to an ACE2, in a specific redox state and conformation, and only in the absence of Ang II or apelin substrates. It is unclear how dimeric and/or monomeric forms of ACE2 influence the activity of B^0^AT1 and SIT1/SGLT1 in response to the binding of one or two SARS-CoV-2 particles in patients with COVID-19. Elevated levels of Ang II are reported in COVID-19 patients and are directly proportional to viral load [109]. In agreement with the Ang II/ATR1 responses, these patients exhibit high levels of circulating inflammatory markers, such as C-reactive protein (CRP) [109], hence SARS-CoV-2 binding to ACE2 results in the prolonged, ‘locked’ state governed by high levels of Ang II (Figure 2C). The mortality was higher in patients using ACE inhibitors and AT1R blockers (36.8%) than in patients without their use (25.6%) [110], suggesting that at this stage of pathogenesis, Ang II may compete with SARS-CoV-2 for binding to ACE2, thereby decreasing viral load. However, a larger study [111] found similar mortality in patients with or without ACE inhibitors/AT1R blockers. Proapelin and apelin levels were not investigated in COVID-19 patients, and their contribution to lung inflammation and injury remains unknown. We will extrapolate the contribution of ACE2 complex to pathogenesis based primarily on the clinical data reported in COVID-19 trials and mechanistic studies in the model systems.

SARS-CoV-2 appears to disrupt the assembly of the B0AT1/SIT1/SGLT1 complex with ACE2 for the absorption of aa and glucose. In one third of transgenic mice expressing human ACE2 [112], 30% t-hACE2 mice lost 20% of initial weight within 2–6 days after SARS-CoV-2 infection [112]. COVID-19 patients, both lean and obese, also rapidly lose −36.9% and −24.4 % fat mass, respectively, accompanied by wasting of lean mass during their 20 days of hospitalization [113]. Although inflammation and hospitalization stress are canonic contributors to wasting syndrome considered in this study [113], malabsorption of glucose and amino acids by B0AT1/ SIT1/SGLT1 complex in the presence of SARS-CoV-2 could underlie wasting. Diarrhea, vomiting [114], anosmia, ageusia and dysphagia [115] could augment weight loss in patients with SARS-CoV-2 infection. Neutral aa may alleviate these symptoms. ACE inhibitors improve the expression of both Ace2 and Slc6A19, as well as other amino acid Slc36A1 and Slc15A1 transporters in the intestinal tissues in treated vs. untreated patients [80]. Whether ACE inhibitors provide a therapeutic remedy for rapid weight loss in COVID-19 patients remains to be determined. Other pro/con considerations of ACE and ATR1 inhibitors were recently discussed [116]. Hijacking of the ACE2 complex is a key feature shared by pathogenic SARS-CoV, HCoV-NL63, and SARS-CoV-2; however, severe pathogenicity and infectivity coincide with the acquisition of new structural sequences by SARS-CoV-2 [21] and the ability to facilitate interaction with TMPRSS2 [117].

### 6.1. Structural Site of SARS-CoV-2 and Hyperactivation of ENaC

A notable difference in SARS-CoV and SARS-CoV-2 is a multibasic sequence that is homologous to the furin cleavage site on the αENaC ectodomain [21]. This near-identical furin site enhances SARS-CoV-2 cleavage for replication [104,106] and implies competition between it and the hosts’ αENaC in endosomes for furin-mediated cleavage. Furin is a physiological activator of ENaC [108]. In the absence of furin, ENaC is cleaved on different sites [118,119] by extracellular proteases, including channel-activating protease (CAP1) [120], CAP2/TMPRSS4, CAP3/matriptase, and plasmin [119] (reviewed in [55]), resulting in overactive ENaC. Under inflammatory conditions, this pathway can resolve edema in a mouse model of acute respiratory distress syndrome (ARDS) [119]. ENaC overactivation is promoted by Ang II and leads to loss of K^+^ and an increase in TMPRSS2 expression, which inhibits ENaC (Figure 4, physiological scenario). SARS-CoV-2 employs both furin and TMPRSS2 proteases for its S1/S2 cleavage and subsequent fusion with the host membrane [104,106]. The SARS-CoV-2 sequence mimicking αENaC [21] can potentially bind TMPRSS2, thereby, alleviating inhibition of αENaC by TMPRSS2 [46] (Figure 4, pathological scenario). This arrangement will lock ENaC in cute respiratory distress syndrome a hyperactivation state, ensuring TMPRSS2 expression and redirection of its function for SARS-CoV-2 cleavage. Together, these events can multiply the efficacy of SARS-CoV-2 invasion and cause severe damage to host tissues by hyperactive ENaC.

### 6.2. Evidence of Osmotic Crisis in COVID-19

Transport of Na^+^ by ENaC [46] determines the volume of airway surface fluid in lungs and K^+^ concentration in blood (Figure 4) (excellently reviewed in [32]). The replication of SARS-CoV-2 in 85% of severely ill patients was associated with profound <3–3.5 mmol/L hypokalemia [121], which was confirmed using a weighted mean difference from five pooled studies [122]. The levels of K^+^ were proportional to the severity of disease measured by body temperature, CRP, and other factors [121]. Hypokalemia occurs despite supplementation with K^+^ [121] and was accompanied by hyponatremia and hypocalcemia: only Cl^−^ levels were within physiological range [122]. The hypoalbuminemia in COVID-19 patients [121] suggests a change in Donnan equilibrium, due to imbalance in the intracellular organic osmolytes and Na^+^. Although mechanistic studies would provide analysis of these phenomena in the future, these clinical findings are consistent with hyperactivation of ENaC. In fact, lung weight among patients who died from influenza A-induced ARDS was 230% heavier than lung weight in COVID-19 patients (161%), compared to the lung weight in age-matched control group patients without ARDS (100%) [20]. In influenza A-infected cells, inflammatory cytokines induce vascular permeability and extracellular efflux of Na^+^ and water [59]. In contrast, hyperactive ENaC, which mediates influx Na^+^ and water into lung epithelium, is a probable factor accounting for differences in fluid volume in these lungs.

In lung alveolar cells, organic osmolytes resolve osmotic instability. SGLT1 is expressed in lungs, where it reabsorbs Na^+^ and glucose from the airway surface liquid [123], probably in conjunction with the ACE2 complex (Figure 5B). This function is disrupted in diabetes, and increases the predisposition of patients with diabetes to bacterial infections. Patients with type 2 diabetes infected with SARS-CoV-2 appear to rapidly develop ketoacidosis on standard anti-diabetic medication [124], suggesting impaired influx and deficient intracellular concentrations of glucose. These findings are in agreement with dysfunction of the ACE2/B0AT1/SIT1/SGLT1 complex in the lungs of patients with COVID-19 [123,125]. The characteristics of permanent unresolved osmotic stress in cells include initiation of reversible thrombosis increasing D-dimer levels in blood, necrosis in ATP-deprived cells, or cell rupture causing acute thrombosis and inflammation. In patients with SARS-CoV-2 infection, increase in D-dimer levels (>2 µg/mL) at hospital admission accurately (92% sensitivity and 83% specificity) predicted mortality from thrombotic events [126]. D-dimer increase is validated in many COVID-19 patients worldwide [127,128]. Although, viral particles were found primarily (90%) in pneumocytes [50], while endothelial cells expressing ACE2 have no viral particles (0%) [50]. Nonetheless, microthrombosis affects capillaries in multiple organs leading to thrombotic microangiopathy [129] and deep vein thrombosis [130] in COVID-19 patients. These findings support that systemic factors, such as change in cellular osmolality, is implicated in thrombotic events. Arguably [19], venous multiorgan microthrombosis is the most distinguishing characteristic of COVID-19 compared to other viral pathologies. 

Necrosis in type 2 pneumocytes occurred in all studied COVID-19 patients [50]. Finally, cell rupture was evident from histological examinations of pulmonary capillaries [20] as well as the elevated marker of cell damage LDH, which affected 60% of COVID-19 patients. Myocardial damage also developed in 7–17% of COVID-19 patients and its progression to myocarditis is responsible for 7% of deaths (reviewed in [131]). The levels of troponin, a marker of myocardial cell damage, correlated with the severity of COVID-19, and mortality levels [110]. These manifestations are consistent with prolonged loss of osmotic balance, but distinct from other viral infections leading to apoptosis induced by inflammation and ROS. Striking differences in ADRS pathogenesis [20] were revealed after direct comparison of histologic analysis of pulmonary vessels from seven patients who succumbed to SARS-CoV-2 or influenza A (H1N1) infections. ACE2 expression was markedly ~10-times reduced in both alveolar and capillary endothelial cells in COVID-19 compared to influenza patients. Microthrombosis was 9-times more prevalent in SARS-CoV-2 than in influenza A(H1N1) pulmonary capillaries [20]. The expression of inflammatory factors IL6, CXCL10, TNFRSF1A, VCAM1 as well as hypoxia transcription factor HIF1A and HMOX1, and IGF1 were similar in both patient groups [20], suggesting specific prothrombotic mechanisms. Combined findings highlight that osmotic crisis involving overactivation of ENaC and suppression of the ACE2 complex can be a critical mechanism of thrombosis.

### 6.3. Osmotic Instability as a Risk Factor for Severe COVID-19 Pathogenesis

A perplexing feature of COVID-19 is the severity of pathogenesis in different patients ranging from asymptomatic to ARDS augmented by thrombosis and multiorgan failure [132,133]. SARS-CoV infections had similar, although less pronounced manifestations [134]. SARS-CoV-2 pathogenesis is more severe in persons of older age, male sex, and with hypertension, diabetes, obesity, and cardiovascular disease (reviewed in [19]). These metabolic conditions are linked to decreased ACE2 levels and functions in different tissues [135] (reviewed in [18]). Although low levels of ACE2 [135] expect to decrease the risk of SARS-CoV-2 infection, 75–85% of hospitalized patients were above the age of 50 years [19,133]. This higher morbidity and mortality in the aged population is congruent with the increased number of senescent cells with deregulated cell volume as a result of aging [136]. Hypertension, obesity, and diabetes also have preexisting deregulation of the RAS system [18]. Moreover, in diabetes, the influx of the major organic osmolyte glucose is already compromised, and the hijacking of remaining ACE2 by SARS-CoV-2 could facilitate osmotic collapse. Recently, genome-wide association analysis was performed in more than 1500 COVID-19 patients with ARDS and respiratory failure and population-based controls [137]. A significantly higher frequency of the risk allele for severe COVID-19 was found on chromosome 3, which codes for the SIT1 amino acid transporter as well as receptors for inflammatory cytokines, CC-motif chemokine receptor 9 (CCR9) and the C-X-C motif chemokine receptor 6 (CXCR6) among other genes [137]. This finding highlights an importance of the ACE2 partner SIT1 in the development of severe symptoms of COVID-19. Addressing intracellular tonicity could provide a missing link preventing microthrombotic complications, respiratory malfunction, and multi-organ failure. 

### 6.4. Sensory and Neuronal Interplay of ENaC, ACE2, and TMPRSS2 in COVID-19

ENaC-dependent cell polarization plays a central role in the regulation of sensory input in neurons. Physiological ENaC levels in epithelial membranes were implicated in sensing the taste of salt and acid [138,139,140,141] transduced by gustatory chorda tympani (CT), which also express AT1R [139]. Na^+^ influx in tastebuds elicits suprathreshold depolarization activating voltage-gated neurotransmission by calcium homeostasis modulator 1 and 3 (CALHM1/3) [142]. Deficiency in αENaC abolishes salt taste in mice [143]. Stimulation with Ang II [144] or inflammatory mediators TNFα and LPS [145], reversibly decreases salt taste sensitivity in an activated ENaC-dependent fashion, even though other voltage-dependent and independent mechanisms for Na^+^ influx are also involved [145]. The mechanism underlying odor sensitivity is less clear, even though TMPRSS2, ACE2, and ENaC are expressed in nasal epithelial cells [48,146] in olfactory epithelium and, to a lesser extent, in olfactory neurons in an age-dependent manner [147]. ENaC amplifies the perception of odors, at least, in drosophila [146,148]. TMPRSS2, ACE2, and ENaC are also expressed in the tongue [138,149,150]. Hyperactivation of ENaC may present a plausible effector mechanism for altered sensory perception during stress. 

Neurons of mammalians with high cognitive performance express δENaC in all regions in the brain, particularly in the cerebellum and hippocampus, as well as heart, and pancreas [151]. Low pH activates δENaC, which triggers H^+^ influx and a current, resulting in neuron desensitization [151] and ATP secretion [152]. EDTA, hypo-osmolarity, and lactate δENaC augment this response, suggesting its upregulation under hypoxic and/or inflammatory conditions [153]. In humans, αβγδENaC influences presynaptic plasticity resting membrane potential and excitability of neurons [154,155]. Aldosterone also activates ENaC in neurons [156] by PKC, which negates the actions of glucocorticoid-induced kinase 1 [157]. ENaC activity generates tonic changes in membrane potential responsible for basal firing in vasopressin and oxytocin neurons [155], and contributes to the neuroregulation of hypertension [158]. Despite evidence of ENaC’s role in control of fundamental neuronal functions, in vivo data remains scarce. Activation of ENaC in haploinsufficient Nedd4L^−/−^ mice [159] was associated with hyperactivity and increased sensitivity to inflammation-associated pain; however, neither ENaC nor its co-activated Cav3.2 calcium channel [160] were examined in this study. TMPRSS2 is also expressed at low levels in the brain, particularly in the pituitary gland and cerebellum [161] that also express αβγδENaC and ACE2. Cleaved forms of TMPRSS2 bind to protease-activated receptor 2 to induce Ca^2+^ influx, which regulates pain and allodynia in patients with cancers expressing TMPRSS2 [162]. The role of TMPRSS2 in the brain remains unknown. ACE2 is enzymatically active in the human brain [163] and its impact on anxiety-like behavior mediated by AngII/AT1R [164]. Overexpression of *Ace2* altered the frequency of spontaneous inhibitory postsynaptic currents regulating GABA release onto GABAergic neurons in the basolateral amygdala [165]; however, the dependence of these currents on ENaC remains to be determined. Inhibition of Ang 1–7/MasR abolishes this effect [165]. ACE2 also decreases stress-activated hypothalamic-pituitary-adrenal (HPA) axis in males [166], but not in females [167]. Thus, catalytically active ACE2 switches anxiogenic effects of Ang II to anxiolytic responses of Ang 1–7 by an effector mechanism modulating postsynaptic currents. 

SARS-CoV has been detected in the brain [168] and SARS-CoV-2 invasion has been proposed based on an in vitro study [169]. Recent study reports the presence of SARS-CoV-2 in the brains of transgenic mice that overexpress human ACE2, and in human cortical neurons post-mortem [170]. Cantuti-Castelvetri et al. have proposed an alternative neurophilin-1 (NRP1) pathway for SARS-CoV-2 entry into the sensory epithelium and, possibly, neural tissues [171]. In this study, S protein was detected in the olfactory epithelium and neuronal progenitors in five of the six examined tissues isolated from COVID-19 patients post-mortem [171]. However, in the absence of viral mRNA expression in these cells, the replication efficacy of SARS-CoV-2 in the context of NRP1 remains unclear. Interestingly, the detection of SARS-CoV-2 has been challenging [170,171], although patients infected with SARS-CoV-2 manifest neurological symptoms at early onset and throughout COVID-19 pathogenesis [172,173]. An array of neurological symptoms was provoked by SARS-CoV infections (reviewed in [172,173]). While host machinery, including ACE2 and TMPRSS2, supports the similar entrance [99,102], inflammation, and hypoxia across coronavirus infections (reviewed in [174]), neurological manifestations in sensory and nervous tissues are distinct in COVID-19 [175,176]. Noteworthy, COVID-19 symptoms may be resultant of not only direct invasion of CNS, but also collateral damage of respiratory and cardiovascular systems, or neurohypertension [177]. The important difference between SARS-CoV-2 and SARS-CoV is also an ENaC-mediated polarization in sensory cells and neurons. Self-reported data collected in a cross-sectional study reports the loss of smell (anosmia) and taste (hypogeusia) that preceded the onset of the systemic and more severe disease symptoms [176,178] (reviewed in [179]). These observations agree with the role of ENaC in the mediation of salt and acid taste [139]. Polyneuritis cranialis or convulsions in COVID-19 patients [172,173] can reflect ENaC and TMPRSS2 malfunctions in trigeminal neurons. The anticipated impact of SARS-CoV-2 intercepting ACE2, TMPRSS2, and ENaC could provide a mechanistic cue to severe anxiety and increase in suicidal tendencies in COVID-19 patients [180], which are commonly attributed to isolation during hospitalization. Future investigations will determine the requirements for the treatment of these conditions. As the syndromic complexity of SARS-CoV-2 infection continues to evolve, the collection of data documenting electrolyte balance may unravel mechanisms of sensory and neuronal damage.

## 7. SARS-CoV-2 Furin Cleavage Site and Infectivity

The cleavage of S1/S2 proteins at the acquired furin site [106] has been proposed to explain an increase in infectivity of SARS-CoV-2 vs. SARS-CoV [104,107]. This cleavage elicits fusion of a SARS-CoV-2 infected cell with an uninfected cell, forming syncytium [104]. The formation of filopodia containing processed SARS-CoV-2 has been shown to propagate infection in a Caco2 cell culture by casein kinase 2-dependent activation of the cytoskeleton [181], whereas the relation to furin was not examined. Furin-mediated cleavage was sufficient to propagate infection in pulmonary cells; however, TMPRSS2 and other proteases can facilitate fusion of infected and uninfected cells [104]. SARS-CoV-2 predominantly invades pneumocytes (90%) and is present to a minor extent in other cell types [50]. Infected pneumocytes were distributed peripherally and involved upper and middle lung lobes, according to multiple studies comparing pneumonia in COVID-19 with other viral pneumonias [182]. This pattern of pneumonia in COVID-19 patients appears to be in support of cell-to-cell transmission [104] rather than the exposure of multiple cells to SARS-CoV-2. It remains to be investigated if cell-to-cell infection is responsible for the long, 14-day incubation period prior to the manifestation of COVID-19 symptoms.

Both furin inhibitors [104] and CK2 inhibitors [181] were suggested to prevent cell-to-cell SARS-CoV-2 infections, although serious side effects are anticipated from these unspecific therapies. Regulation of cholesterol content in membranes can be another way to control furin activity and ACE2 shedding. SARS-CoV and ACE2 reside in cholesterol-rich [183] lipid rafts, which elicit their endocytosis in the host cell [183]. Cholesterol-enriched rafts are also an obligate environment for furin-mediated cleavage of different substrates, including ADAM17 and collagen XXIII [184,185]. The loss of cholesterol directly impacts inflammation and induces ACE2 shedding by ADAM17 [184,186]. Although the extent of furin inhibition and facilitation of ACE2 shedding in a SARS-CoV-2 infected cells relative to other proposed mechanisms [187,188] needs to be examined further, a retrospective study demonstrated reduction of all-cause mortality from 9.4% to 5.2% in COVID-19 patients receiving cholesterol-lowering therapy with statins [189]. Other studies confirm this effect [190]. The efficacy of intervention involving delivery of statins directly into lungs vs. systemic inhibition of cholesterol synthesis requires further investigation. 

The therapeutic inhibition of TMPRSS2 is commonly proposed as a strategy to prevent entry of viruses that require serine protease cleavage [102,191]. Protease inhibitors, including camostat mesylate, gabexate, and aprotinin, predominantly have a broad range of specificity [102,191,192] and elicit notable antifibrinolytic side effects [193]. The lack of any pathological manifestations in *TMPRSS2*^−/−^ mice [194] draws interest to therapeutic inhibition of TMPRSS2 as a possible safe target to decrease SARS-CoV-2 transmission [102]. The evidence that hypokalemia triggers expression of TMPRSS2 suggests potassium supplementation as a promising strategy to prevent loss of K^+^, lung hypertension, normalize cell volume, and prevent TMPRSS2-dependent viral infectivity.

## 8. Conclusions

The evolutionary success of viruses is determined by their ability to intercept critical signaling hubs of the host organism, which are centered on a multifunctional protein with a remarkable capacity to interact with other partner proteins governing host metabolism and immunity. The unprecedented morbidity of SARS-CoV-2 is based on its ability to occupy two hubs. The interception of ACE2 hub (Figure 5) is accompanied by overproduction of Ang II and destabilization of glucose and amino acids’ transport for nutritional and osmotic functions, that are required to resolve inflammation. The structural decoy-site on SARS-CoV-2 [21] enables an overactive state of host ENaC that further disrupts local and systemic osmotic and ionic homeostasis, as well as polarization in neurons, barrier, and sensory cells (Figure 4). Experimental and clinical studies need to validate this mechanism. However, the presence of E protein on all beta coronaviruses, which forms an ion channel with 10-fold higher permeability for Na^+^ than for K^+^ ions [195], suggests that regulation of ion concentrations is a fundamental requirement for replication of these pathogens. Hijacked together, ACE2 and ENaC hubs collapse cellular osmolality in COVID-19 patients that leads to ATP depletion and necrotic death or hypotonic rapture releasing LDH [196], microthrombosis [197], and hypoxia. Alone, therapeutics reducing Ang II levels and AT1R responses cannot alleviate SARS-CoV and SARS-CoV-2 complications [19]. The combinatorial onset of this osmotic crisis requires combinatorial therapies as effective solutions.

## 9. Therapeutic Perspectives

Arguably, the most important advantage of the acquired furin site is in redirecting TMPRSS2 function for proteolytic cleavage of SARS-CoV-2 (Figure 2C), opening a possibility of cell-to-cell propagation of infection [102,104] in seemingly asymptomatic patients [198] (reviewed in [19]). This special type of pathogenesis calls for alteration to standard therapeutic measures. COVID-19 patients receiving intravenous remdesivir recover only four days earlier than placebo control and have no reported data on patient survival [199]. Therapies inhibiting viral replication or inflammation reveal marginal efficacy (reviewed in [19]). Based on discussed interactions and infectivity data (reviewed in [19]), furin inhibitors [104] and CK2 inhibitors [181] are expected to be efficient during the first five days of asymptomatic infection. The encouraging decrease in mortality in the statin-treated group of COVID-19 patients [189] suggests that statins are a safe adjuvant therapy that decrease furin output and improve all stages of the disease. The attempt to treat COVID-19 patients with recombinant ACE2 [200] would likely be only partially effective in inhibition of cell-to-cell propagation mediated by TMPRSS2 and furin cleavage [102,104].

Hypokalemia found in COVID-19 patients in ~75% of COVID-19 cases [196] must be addressed in future clinical trials. Although initial potassium supplementation was performed in patients with severe COVID-19 manifestations [121], its impact on TMPRSS2 expression, ENaC activity, LDH levels, blood and urine osmolality, and the rate of viral replication has not been examined to validate the role of TMPRSS2/ENaC axes in the amplification of SARS-CoV-2 propagation and osmotic crisis. Future clinical studies need to be performed in COVID-19 patients including newly infected subjects and patients with severe pathogenesis, to elucidate the role of hypokalemia in increasing *TMPRSS2* expression, which contributes to SARS-CoV-2 propagation and osmotic crisis. Cardiologic manifestations of COVID-19, microvascular and endothelial disease and heart failure [131] have been proposed as the critical mechanisms leading to COVID-19-related mortality [201,202]. The specific underlying mechanisms of endothelial damage beyond inflammation in COVID-19 patients have been established in clinical studies [20]; however, it remains elusive, because SARS-CoV-2 does not replicate in endothelial cells [51]. The osmotic crisis resulting from hypokalemia and disruption of the ACE2-based complex is compatible with clinical findings in damaged endothelial cells, early manifestations of elevated D-dimer levels, and the progression to thrombosis in COVID-19 patients. Current clinical trials use fibrinolytic and anti-inflammatory therapies reviewed in [19] to combat thrombosis. To address osmotic crisis, adjuvant supplementation with neutral amino acids could be implemented to replenish organic osmolytes and stabilize intracellular osmolality when the ACE2 complex is disabled. This safe approach improves the rate of distress, executive functions, attention, and vigilance in patients with genetic disorders and malnutrition [203]. Normalization of hypokalemia, resulting from the hyperactivation of ENaC, could be achieved by a moderate dose of amiloride combined with potassium and neutral amino acids’ supplementation to address both the root problem and its consequences.

More therapeutic options may be developed in the future using recombinant chimeric protein technology. Apelin can compete with the virus for binding to ACE2 and participate in the resolution of inflammation and hypoxia [204]. The soluble TMPRSS2 or a chimeric protein combining the catalytic sites of ACE2, TMPRSS2, and the furin site of ENaC, may serve as a decoy for the virus and mediate its cleavage directly in circulation. Breakthrough in management of hypertension was made following discovery of RAS pathway. COVID-19 pathology prompts us to understand the versatile biology of ACE2-complex and its ENaC counterpart in the regulation of intracellular osmosis that could also shed light on complications of metabolic degenerative diseases, such as aging and diabetes. The most remarkable feature of COVID-19 is the range of host responses to the infection [132,133] indicating that the host response is the main culprit in morbidity and mortality. Decipherment of dynamic interactions among proteins within host signaling hubs may be the ground for development of effective therapies for infectious and degenerative metabolic diseases.

## 10. Patents

There are no patents resulting from the work reported in this manuscript.

## Abbreviations

AA	Amino Acids
ACE	Angiotensin-converting enzyme
ACE2	Angiotensin-converting enzyme 2
ADAM17	Disintegrin and metalloproteinase 17 (Alias: TACE)
AMPK	Adenosine monophosphate-activated protein kinase)
Ang II	Angiotensin II (Alias: Ang 1–8)
Ang (1–10)	Angiotensin I
AP1	Activator protein 1 transcription factor
AQP1	Aquaporin 1, a water channel protein
AT1R	Angiotensin II Type 1 Receptor
APJ	Apelin receptor
ARDS	Acute Respiratory Distress Syndrome
ASCT2	Alanine, serine, cysteine-preferring transporter 2 (gene SLC1A5)
AVP	Arginin vasopressin
B0AT1	B degrees amino acid transporter 1 (gene SLC6A19)
CALHM1/3	Calcium homeostasis modulator 1 and 3
CAM	Calmodulin
CAMKII	CaM-dependent kinase
CAP1	Channel-activating protease
CatA	Cathepsin A
CK2	Casein kinase 2
CNS	Central nervous system
COX2	Cyclooxygenase 2
COVID-19	Coronavirus Disease of 2019
CRP	C-reactive protein
CT	Chorda tympani
DAG	Diacylglycerol
ECM	Extracellular matrix
EDTA	Ethylenediaminetetraacetic acid
ENaC	Epithelial sodium channel
ER	Endoplasmic reticulum
Gαq/11	Guanine nucleotide binding protein (G protein), alpha 11 (Gq class)
GABA	Gamma-aminobutyric acid
GDP	Guanosine diphosphate
GI	Gastrointestinal tract
Glu	Glucose
GLUT1	Glucose transporter 1
GTP	Guanosine triphosphate
HAT	Transmembrane serine protease 11D (Alias: TMPRSS11D)
H1N1	Influenza A
HCoV-NL63	Human coronavirus NL63
HIF1	Hypoxia Induced Transcription Factor
HMOX1	Heme Oxygenase-1
HPA	Hypothalamic-pituitary-adrenal
iAA	Imino acids, e.g., proline
IP3	Inositol 3 phosphate
LDH	Lactate dehydrogenase (Alias: Lactic acid dehydrogenase)
LPS	Lipopolysaccharide
MAS	MAS1 proto-oncogene, G protein-coupled receptor
mTORC1	Mammalian target of rapamycin complex 1 (Alias: Mechanistic target of rapamycin complex 1)
nAA	Neutral amino acids
NFAT5	Nuclear Factor of Activated T cells (Alias: TonEBP, tonicity-responsive enhancer binding protein)
NHE3	Sodium–hydrogen exchanger 3
NO/iNOS	Nitric Oxide
ORF8	Envelope glycoprotein B (Gene: ORF8)
PAI-1	Plasminogen Activator Inhibitor-1
PKC	Protein kinase C
PLC	Phospholipase C
RAS	Renin-Angiotensin System
RBD	Receptor binding domain
ROS	Reactive Oxygen Species
sACE2	Soluble angiotensin-converting enzyme 2
SARS-CoV	Severe Acute Respiratory Syndrome Coronavirus
SARS-CoV-2	Severe Acute Respiratory Syndrome Coronavirus 2
SGK1.1	Glucocorticoid-induced kinase 1
SGLT1	Sodium-dependent Glucose Transporter 1
SIT1	Sodium/Imino-acid Transporter 1 (gene: SLC6A20)
SLC6A19	Solute Carrier Family 6 Member 19 (protein BoAT1)
TACE	Tumor Necrosis Factor-α Converting Enzyme (Alias: ADAM 17)
TAPI2	TNF Protease Inhibitor 2
TMEM27	Transmembrane protein 27 (Alias: Collectrin, Gene: CLTRN)
TMPRSS2	Transmembrane Serine Protease 2
VP	Vasopressin
vWF	von Willebrand factor
WT	Wild type
